# Cancer and fertility management: FIGO best practice advice

**DOI:** 10.1002/ijgo.70426

**Published:** 2025-07-31

**Authors:** Nikhil Purandare, Francisco Ruiloba, Long Nguyen‐Hoang, Sarikapan Wilailak, Nozomu Yanaihara, Jacqueline P. W. Chung, Jaideep Malhotra, Edgar Mocanu, Frédéric Amant, Orla McNally, Inge Peters, Katie Flynn, Aya El Helali, Cynthia Maxwell, Sumaiya Adam, Lina Bergman, Surabhi Nanda, Valerie Tiempo Guinto, Fionnuala M. McAuliffe, Catherine Nelson‐Piercy, Melanie Nana, Graeme Smith, Jonathan Berek, Sharleen O'Reilly, Pat O'Brien, Bo Jacobsson, Liona C. Poon, the FIGO Committee on the Impact of Pregnancy on Long‐term Health and the FIGO Division of Maternal and Newborn Health and in collaboration with the FIGO Committee on Women's Cancer and the FIGO Committee on Reproductive Medicine, Endocrinology and Infertility

**Affiliations:** ^1^ Department of Obstetrics and Gynecology University Hospital Galway Ireland; ^2^ Fetal Medicine Research Unit King's College Hospital London UK; ^3^ Fetal Medicine Centre Tam Anh HCMC General Hospital Ho Chi Minh City Vietnam; ^4^ Department of Obstetrics and Gynaecology Prince of Wales Hospital, Chinese University of Hong Kong Hong Kong SAR China; ^5^ Faculty of Medicine, Ramathibodi Hospital Mahidol University Bangkok Thailand; ^6^ Department of Obstetrics and Gynecology The Jikei University School of Medicine Tokyo Japan; ^7^ Rainbow IVF Agra Uttar Pradesh India; ^8^ Department of Obstetrics and Gynecology Rotunda Hospital Dublin Ireland; ^9^ Division of Gynecologic Oncology UZ Leuven Leuven Belgium; ^10^ Department of Obstetrics and Gynaecology Royal Women's Hospital Melbourne, University of Melbourne Melbourne Australia; ^11^ Department of Woman's and Child Health and Public Health Sciences, Gynaecologic Oncology Unit Fondazione Policlinico Universitario Agostino Gemelli IRCCS Rome Italy; ^12^ Department of Clinical Oncology Centre of Cancer Medicine, Hong Kong University Hong Kong SAR China; ^13^ Maternal Fetal Medicine Women's College Hospital and Mount Sinai Hospital Toronto Ontario Canada; ^14^ Department of Obstetrics and Gynaecology School of Medicine, Faculty of Health Sciences, University of Pretoria Pretoria South Africa; ^15^ Diabetes Research Centre, Faculty of Health Sciences University of Pretoria Pretoria South Africa; ^16^ Department of Obstetrics and Gynecology Institute of Clinical Sciences, Sahlgrenska Academy, University of Gothenburg Gothenburg Sweden; ^17^ Guy's and St Thomas NHS Foundation Trust London UK; ^18^ University of the Philippines‐Philippine General Hospital Manila Philippines; ^19^ Department of Obstetrics and Gynecology Asian Hospital and Medical Center Muntinlupa Philippines; ^20^ St Luke's Medical Center–Global City Taguig Philippines; ^21^ Perinatal Research Centre University College Dublin, National Maternity Hospital Dublin Ireland; ^22^ Division of Obstetric Medicine King's College London London UK; ^23^ Department of Obstetrics and Gynecology Kingston Health Sciences Centre, Queen's University Kingston Ontario Canada; ^24^ Stanford Women's Cancer Center, Stanford Cancer Institute, Stanford University School of Medicine Stanford California USA; ^25^ School of Agriculture and Food Science University College Dublin Dublin Ireland; ^26^ University College London Hospitals London UK; ^27^ Department of Obstetrics and Gynaecology Region Västra Götaland, Sahlgrenska University Hospital Gothenburg Sweden; ^28^ Department of Genetics and Bioinformatics, Division of Health Data and Digitalisation Institute of Public Health Oslo Norway

**Keywords:** adolescents and young adults, cancer, fertility preservation, gonadotoxicity, oncofertility, patient counseling, reproductive health

## Abstract

Cancer diagnoses in patients of reproductive age require balancing urgent oncological treatment with the need to preserve fertility. This FIGO Best Practice Advice outlines key considerations for fertility management in this population given the rising cancer incidence among young women and the reproductive risks posed by cancer treatments. The guidance evaluates the impact of chemotherapy, radiotherapy, surgery, and emerging therapies—such as targeted agents and immunotherapies—on gonadal function and fertility. Established fertility preservation methods, including oocyte/embryo cryopreservation, sperm banking, and ovarian tissue freezing, are detailed alongside barriers to their adoption, such as cost and limited access. Early collaborative counseling with oncologists and fertility specialists is central to addressing timelines, psychological impacts, and priorities. Post‐treatment pathways, including assisted reproduction and surrogacy, are also explored. The guidance stresses the importance of integrating fertility‐sparing interventions and fertility preservation into cancer care while advocating for equitable access to resources. Further research is needed to refine preventive interventions, evaluate long‐term outcomes, and expand options for survivors globally. By prioritizing fertility preservation within oncological care, healthcare providers can better support the holistic needs of young individuals facing cancer.

## 1 INTRODUCTION

For adolescents and young adults facing a cancer diagnosis, the fight for survival is often intertwined with concerns about their future, particularly the ability to have children. Among women of reproductive age, approximately 1 million cancers are diagnosed every year.^1^ In 2021, over 383 000 new cancer cases were diagnosed in women aged 15–39 years globally, causing over 81 000 deaths. Epidemiological data indicate a concerning rise in incidence among this demographic over the past three decades.^2^ Adolescents and young adults (15–39 years) face a disproportionate burden, with an incidence twice as high in women (52.9 vs. 28.3 per 100 000) and higher mortality (13.1 vs. 10.6 per 100 000) compared with men in 2022.^3^

The most common cancers in adolescents and young adults include breast, cervical, ovarian, endometrial, thyroid, and colorectal cancers, as well as leukemia, lymphomas, and melanoma.^2^, ^3^, ^4^ Cancer treatments, including chemotherapy, radiotherapy, and surgery, can pose a substantial threat to fertility by causing premature ovarian failure and diminished ovarian reserve. In affected males, these treatments can result in azoospermia.^1^, ^5^ For young patients, the ability to conceive and give birth is critical for quality of life and long‐term well‐being, making fertility preservation an essential component of comprehensive cancer care.^6^

Chemotherapy, particularly alkylating agents, can severely impact female fertility by damaging ovarian follicles, with the risk of infertility increasing with age and specific drugs used; cyclophosphamide for breast cancer is notably linked to premature ovarian insufficiency.^7^ While younger women are less likely to experience permanent amenorrhea, they still face a heightened risk of premature menopause, even if menstruation resumes after treatment.^8^ Radiation therapy also threatens fertility, especially when the pelvic region is targeted. The extent of ovarian damage depends on the dose and treatment area. Pelvic radiation can also impair uterine function through fibrosis and reduced myometrial elasticity, raising the risk of miscarriage, preterm birth, and low birth weight—even when ovarian function remains intact.^9^ The increasing incidence of young‐onset colorectal cancer presents unique challenges concerning treatment and reproductive function.^10^ Recognizing that fertility concerns are often second only to survival for these young individuals underscores the necessity of integrating fertility preservation into oncological care.^10^

Rapidly evolving cancer therapies, particularly molecularly targeted agents and immunotherapies, have transformed the prognosis for many patients with solid tumors. However, these advancements introduce challenges for young individuals of reproductive age, creating uncertainties about their long‐term fertility. Immune checkpoint inhibitors (used widely in melanoma and lung cancer) have unclear effects on reproductive tissues. While they enhance antitumor immunity, their potential to induce autoimmune reactions in endocrine organs, such as the ovaries or testes, raises theoretical concerns about hormonal dysfunction or impaired gametogenesis.^6^ Similarly, antibody–drug conjugates like trastuzumab emtansine, which deliver cytotoxic payloads to cancer cells, may inadvertently affect healthy gonadal cells, although clinical data remain sparse.^6^ Small‐molecule inhibitors targeting oncogenic pathways, such as epidermal growth factor receptor (EGFR), anaplastic lymphoma kinase (ALK), or poly (ADP‐ribose) polymerase (PARP), often require long‐term use, increasing the risk of cumulative damage to reproductive organs. For example, PARP inhibitors, commonly used in cancers with breast cancer gene (BRCA) mutations, may exacerbate ovarian insufficiency in premenopausal women. Monoclonal antibodies like bevacizumab, which are contraindicated during pregnancy due to teratogenic effects, lack robust data on pretreatment gonadal toxicity.^6^ Moreover, the safe washout period for many of these newer agents before conception remains largely undefined, adding another layer of complexity for family planning.

Current fertility preservation methods, including oocyte/embryo cryopreservation, sperm banking, and ovarian tissue freezing, remain cornerstone options. However, their integration into care pathways for patients receiving novel therapies is inconsistent. Emerging techniques, such as in vitro follicle maturation or testicular stem cell preservation, offer hope but require validation. A critical gap exists in understanding how these interventions interact with targeted therapies. For instance, ovarian stimulation protocols may need adjustment for patients on tyrosine kinase inhibitors (TKIs), while the timing of tissue cryopreservation relative to immunotherapy cycles remains unexplored. Despite increasing availability, significant barriers persist, including limited awareness among healthcare providers, time constraints before treatment initiation, and inequitable access to fertility preservation technologies.^11^, ^12^ Given the potential impact of cancer treatments on fertility, various preservation options are available for both men and women. For women, embryo cryopreservation is a well‐established method that involves creating embryos from harvested eggs and sperm, which are then frozen for future use.^13^ Oocyte cryopreservation allows for the harvesting and freezing of eggs, providing an option for women who may not have a partner at the time of treatment. Ovarian tissue cryopreservation involves removing and freezing ovarian tissue, which can potentially be reimplanted after cancer treatment. Additionally, the use of gonadotropin‐releasing hormone agonists (GnRHa)—although their protective effect on ovarian function has been most robustly demonstrated in breast cancer patients^14^—may help suppress ovarian activity during chemotherapy. For men, sperm banking is the most common and effective method of fertility preservation, allowing them to freeze sperm prior to treatment for future use. In cases of azoospermia, testicular sperm extraction (TESE) can be performed to retrieve sperm directly from the testes.^15^

The psychological impact of cancer and subsequent fertility concerns can be profound for young survivors. Early individualized discussions about reproductive risks at diagnosis are crucial, as even a single chemotherapy cycle can have detrimental effects.^5^ Early comprehensive counseling—ideally before starting cytotoxic therapy—enables patients to understand their risks and make informed, values‐based decisions.^7^

The intersection of cutting‐edge cancer therapies and reproductive health demands a proactive, patient‐centered approach. While survival gains are paramount, preserving fertility and addressing reproductive concerns are integral to holistic care. Clinicians must address knowledge gaps openly and support research into gonadal toxicity, pregnancy outcomes, and long‐term safety. Embedding oncofertility into routine care empowers young patients to pursue both life and parenthood. As cancer survival rates continue to improve, addressing fertility issues becomes increasingly critical for adolescent and young adult cancer survivors. Healthcare providers play a vital role in guiding patients through the complexities of oncofertility, ensuring access to necessary resources and support to navigate reproductive health after a cancer diagnosis.

## 2 PATIENT COUNSELING

Beyond immediate medical needs and emotional strain, a cancer diagnosis can severely impact fertility. The narrow timeframe for fertility preservation often conflicts with the urgency of cancer treatment, making timely, collaborative counseling essential. This counseling, ideally provided at diagnosis before treatment, should involve oncologists, reproductive endocrinologists, and fertility specialists to ensure personalized patient support.^16^, ^17^

Balancing urgent cancer therapy with the limited window for fertility preservation necessitates compassionate, personalized counseling.^16^, ^17^ Even in highly urgent clinical scenarios requiring immediate therapy, where options may be limited, rapid multidisciplinary coordination remains critical to explore any feasible options, such as emergency sperm banking or ovarian suppression during chemotherapy. This approach aims to integrate treatment with fertility preservation considerations while addressing patient burdens, including financial concerns, and respecting individual parenthood desires.^16^, ^17^

- *Education on gonadotoxicity:* Initiate timely discussions about potential reproductive risks, emphasizing their time‐sensitive nature:
  ⚬ High‐risk therapies: Clearly explain the significant risks of ovarian failure or azoospermia associated with alkylating agents (e.g. cyclophosphamide), pelvic radiation, and total body irradiation.^18^
  ⚬ Emerging therapies: Acknowledge the uncertain long‐term gonadotoxic effects of newer agents such as PARP inhibitors and TKIs, necessitating tailored fertility preservation strategies and ongoing research.^6^
- *Fertility preservation options:* Provide comprehensive information regarding realistic fertility preservation options:
  ⚬ Female patients: Standard options include oocyte, embryo, or ovarian tissue cryopreservation, with success rates influenced by age and ovarian reserve markers (e.g. anti‐Müllerian hormone (AMH) levels). Discuss potential protective role of GnRH agonists, particularly in breast cancer.^5^, ^14^
  ⚬ Male patients: Offer sperm banking as the standard. In cases of azoospermia, inform patients about the availability of TESE.^15^
  ⚬ When gamete collection is not feasible or is unsuccessful, discuss the use of donor gametes and related ethical, legal, and psychological implications, including potential cross‐border treatment and relevant regulations.^19^
- *Preimplantation genetic testing for monogenic disorders (PGT‐M):* Patients with hereditary cancer syndromes (e.g. BRCA, Lynch syndrome) may benefit from PGT‐M to reduce the transmission risk to offspring, requiring coordination with genetic counselors or clinical geneticists.^20^
- *Psychosocial and financial support:* Recognize the significant emotional impact of a cancer diagnosis and the potential for infertility. Counseling should address the psychological and emotional aspects of fertility preservation, ensuring patients have access to mental health resources and relevant support networks. Financial barriers often limit access to fertility preservation options, particularly in low‐resource settings. Therefore, it is crucial to provide transparent information about the costs of fertility preservation and explore available options if appropriate, including insurance coverage, charitable programs, or institutional support.
- *Legal and ethical considerations:* Patients should be informed about the legal frameworks governing fertility preservation in their region, including issues related to ownership and disposition of cryopreserved gametes or embryos, as well as the legal implications surrounding the use of donor gametes.

## 3 EFFECT OF CANCER THERAPIES ON FERTILITY ACCORDING TO DIFFERENT TYPES OF CANCER

The impact of cancer therapies on fertility varies significantly depending on the type of cancer, the treatment regimen, and patient‐specific factors such as age and baseline reproductive health. Table 1 summarizes the fertility risks associated with common cancer types and therapies, as well as the recommended fertility preservation options based on current evidence and international guidelines.^5^, ^21^, ^22^ Factors such as chemotherapy regimen, radiation field and dose, surgical extent, and patient age all contribute to gonadal toxicity.

**TABLE 1:** Fertility risks and preservation strategies by cancer type and treatment.

Cancer type	Common treatments	Fertility impact	Recommended fertility preservation options
Breast	Alkylating agents (cyclophosphamide), ovarian suppression	High risk of POI; cumulative cyclophosphamide dose correlates with amenorrhea rates	Oocyte/embryo cryopreservation, GnRHa during chemotherapy
Cervical	Pelvic radiation, radical hysterectomy	Uterine damage (fibrosis, reduced volume), ovarian failure (if ovaries in RT field)	Ovarian transposition, radical trachelectomy, oocyte/embryo banking
Hematologic (lymphoma)	BEACOPP, ABVD, TBI	BEACOPP: ~80% azoospermia in men, ~50% POI in women; ABVD: Lower risk (≤20% POI)	Sperm banking, ovarian tissue cryopreservation
Pediatric (leukemia)	TBI, alkylating agents (busulfan, cyclophosphamide)	TBI: Near‐100% ovarian/testicular failure in prepubertal patients	Prepubertal ovarian/testicular tissue cryopreservation (controversial due to metastasis risk)
Thyroid cancer	RAI, thyroidectomy	RAI: Potential ovarian damage (dose‐dependent); negligible risk with proper shielding	Oocyte/embryo cryopreservation before RAI
Colorectal cancer	Pelvic RT, oxaliplatin/5‐FU‐based regimens	Gonadal damage with RT; moderate chemo risk (age‐dependent)	Ovarian shielding, ovarian transposition, oocyte/sperm banking
Brain tumors	Cranial RT (≥24 Gy), alkylating agents	Hypothalamic–pituitary dysfunction (50%–80%); gonadal toxicity	Oocyte/sperm banking, growth hormone/FSH/LH replacement
Endometrial cancer	Progestin therapy, hysterectomy	Progestins preserve fertility in 70%–80% of early‐stage cases	Oocyte/embryo cryopreservation before hysterectomy
Sarcoma	Alkylating agents (ifosfamide), pelvic RT	Ifosfamide: High risk of POI/azoospermia; RT: Site‐dependent gonadal damage	Oocyte/sperm banking, gonadal shielding
Testicular cancer	Orchiectomy, BEP (bleomycin, etoposide, cisplatin), RT (rare)	High risk of azoospermia and Leydig cell dysfunction after chemotherapy	Sperm banking (before treatment)

Abbreviations: ABVD, adriamycin, bleomycin, vinblastine, dacarbazine; BEACOPP, bleomycin, etoposide, adriamycin, cyclophosphamide, oncovin (vincristine), procarbazine, prednisone; BEP, bleomycin, etoposide, cisplatin; FSH, follicle‐stimulating hormone; GnRHa, gonadotropin‐releasing hormone agonist; LH, luteinizing hormone; POI, primary ovarian insufficiency; RAI, radioactive iodine; RT, radiation therapy; TBI, total body irradiation.

## 4 FERTILITY OUTCOMES AFTER CANCER TREATMENT

Improved cancer survival often comes at the cost of fertility, which is a key concern for adolescents and young adults. This section explores the factors affecting the postcancer reproductive potential of this population.

### 4.1 Impact of cancer on subsequent pregnancy

Studies involving both childhood cancer survivors and population‐based cohorts have demonstrated a markedly decreased chance of pregnancy.^18^, ^23^, ^24^, ^25^, ^26^ Azoospermia may cause permanent sterility in male survivors without prior sperm banking.^5^ A recent cohort study in Scotland found a 38% reduction in pregnancy likelihood after a cancer diagnosis compared with the general population.^27^ This decrease was consistent across all age groups and cancer types. Beyond conception rates, pregnancies in cancer survivors carry specific risks, with a meta‐analysis showing a 45% higher risk of preterm labor for breast cancer survivors compared with the general population.^28^

FIGO's Best Practice Advice on pregnancy after cancer highlights that survivors, depending on their cancer type and treatment, may face increased risks for various obstetric and perinatal complications, including cardiovascular, endocrine, respiratory, renal, and hepatic issues.^29^

### 4.2 Impact of cancer treatments on fertility

Chemotherapy and radiotherapy may impair fertility based on treatment type, dose, and radiation site. The American Society of Clinical Oncology (ASCO) guidelines classify risk by treatment protocol and patient‐specific factors (e.g. characteristics, drug dosages).^5^, ^30^, ^31^, ^32^ Alkylating agents, for example, reduce primordial follicle counts, with greater ovarian damage in older patients.^33^ Pelvic radiation can damage reproductive organs, including the uterus, leading to scarring and reduced flexibility that may affect future pregnancies. Cranial radiation can disrupt hormonal regulation. The likelihood and timing of ovarian failure depend on ovarian radiation dose.^34^

### 4.3 Estimation of ovarian reserve after cancer

Predicting ovarian function after cancer treatment involves assessing reduced follicle counts using ultrasound and endocrine evaluations of follicle‐stimulating hormone (FSH), AMH, inhibin B, and luteinizing hormone. While AMH has been identified as a clinically useful indicator of gonadotoxicity in young women undergoing cancer treatment,^35^ recent research suggests that oocyte quality, rather than solely AMH levels, may be a critical factor in fertility potential.^36^ Furthermore, it is important to note that AMH levels do not directly and definitively correlate with the chance of spontaneous conception; women with low AMH may still conceive.^36^, ^37^ Accurate fertility prediction in survivorship is crucial, highlighting the importance of fertility preservation strategies like oocyte vitrification and ovarian tissue cryopreservation, especially for individuals at high risk of treatment‐induced infertility. For males, semen analysis is the standard and critical method for assessing fertility status.^5^

## 5 CURRENT OPTIONS FOR FERTILITY PRESERVATION

**FIGURE 1:**
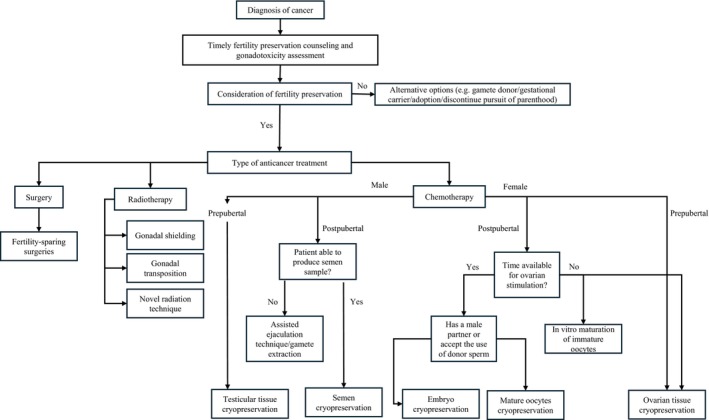
Fertility preservation strategies after cancer diagnosis.

Several options are currently available to help individuals safeguard their reproductive potential (Figure 1 and Table 2).

Oocyte cryopreservation via vitrification provides women, particularly those without a partner, the option to preserve their fertility by freezing their eggs for potential future use.^38^ Even for those in stable relationships, it provides reproductive autonomy, especially given potential relationship changes after a cancer diagnosis. Technological advances have made this process safer and more efficient, with frozen oocytes now demonstrating developmental potential equivalent to fresh oocytes when performed by proficient clinics.^38^ Additionally, antagonist‐based ovarian stimulation can begin without waiting for the menstrual cycle, enabling quicker return to cancer treatment and lowering the risk of complications such as ovarian hyperstimulation.^17^, ^39^

**TABLE 2:** Summary of fertility preservation methods and pregnancy outcomes.^a^

Fertility preservation method	Patient group and timing	Key outcomes and success rates	Major risks and considerations
Male methods			
Sperm cryopreservation	Postpubertal; before therapy (multiple visits)	Gold standard; proven pregnancies and live births	Variable success; requires ART; storage costs
TESE and cryopreservation	Postpubertal (azoospermic); before/after therapy (surgical)	Effective alternative; successful pregnancies with IVF	Surgical risks; requires ICSI; storage costs
TTC	Prepubertal; before therapy (surgical)	Experimental; only option for prepubertal males	Unknown cancer reseeding risk; uncertain reproductive potential
Female methods			
Embryo cryopreservation	Postpubertal; 2–3 weeks (stimulation to retrieval)	LBR: 35%–41%	Requires sperm; delays treatment (2–6 weeks); OHSS risk; ethical concerns
Oocyte cryopreservation	Postpubertal; 2–3 weeks (stimulation to retrieval)	LBR: 26%–32%	Delays treatment (2–6 weeks); OHSS risk
Ovarian transposition	Surgical (no treatment delay)	LBR: 18%–55%; successful pregnancies reported	Rare complications (e.g. bowel obstruction, ovarian cyst formation)
Uterine transposition (experimental)	Surgical (no treatment delay)	Successful pregnancies reported	Uterine ischemia
Conservative gynecologic surgery	Variable timing	LBR: 11%–89%	Recurrence risk (3.1%–15.7%, dependent on cancer type/stage)
Hormonal suppression (GnRHa)	Postpubertal; throughout treatment	May protect ovarian function; limited fertility improvement	Should not replace proven fertility preservation; menopausal symptoms; bone thinning
Ovarian tissue cryopreservation	Prepubertal; No treatment delay (laparoscopic procurement)	LBR: 19%–32%; ovarian function restoration: 70%–95%	Only option for prepuberty; potential malignancy reintroduction
IVM	Variable stimulation/timing	Some live births reported; less effective than mature oocyte/embryo preservation	Limited expertise; low number of births for outcome estimation

^a^ Adapted from Su et al.^5^

Abbreviations: ART, assisted reproductive technology; GnRHa, gonadotropin‐releasing hormone agonist; IVF, in vitro fertilization; IVM, in vitro maturation; LBR, live birth rate; OHSS, ovarian hyperstimulation syndrome; OTC, ovarian tissue cryopreservation; RR, relative risk; TESE, testicular sperm extraction; TTC, testicular tissue cryopreservation.

Embryo cryopreservation can be offered to couples in stable relationships when the male partner can provide a semen sample or if donor sperm is acceptable.^40^ Both partners must agree to the consideration of joint legal ownership of the cryopreserved embryos. Success rates for both oocyte and embryo cryopreservation may vary between assisted reproductive technology (ART) centers. However, in cancer patients, embryo cryopreservation has shown promising results, with live birth rates of around 44%–45%, which is comparable to outcomes in non‐cancer populations.^13^, ^41^

Both oocyte and embryo cryopreservation typically require 2–3 weeks of ovarian stimulation, followed by transvaginal oocyte retrieval where appropriate (e.g. this approach may not be suitable in cases such as cervical cancer). This timeline can delay anticancer treatment, although novel random‐start stimulation protocols can shorten the stimulation process.^39^

In vitro maturation, involving the culture of immature follicles with or without mild gonadotrophin stimulation, offers an alternative approach for patients with limited time for ovarian stimulation. However, this procedure has limited success and lower live birth rates compared with conventional in vitro fertilization (IVF).^42^

Ovarian tissue cryopreservation is particularly important for prepubertal girls or patients who do not have enough time for ovarian stimulation before undergoing high‐risk gonadotoxic therapy. This procedure involves two surgeries: one to harvest the cortical ovarian strips for cryopreservation and another to reimplant the thawed tissue.^43^ A small risk of cancer cell reintroduction exists after transplantation, which varies by cancer type: high in leukemia and lower in many solid tumors, such as breast cancer.^43^ While the overall risk is considered low for many solid tumors, the possibility cannot be entirely excluded, and meticulous histological and potentially molecular evaluation of the cryopreserved tissue is recommended before transplantation.

Pharmacological interventions, such as the use of GnRHa, can also be used in fertility preservation, primarily as a potential adjunct to protect fertility rather than a standalone method. GnRHa induce a menopausal‐like state, which some evidence suggests may help prevent chemotherapy‐induced premature ovarian failure.^40^, ^44^ While a trial in breast cancer patients has demonstrated a doubling of the pregnancy rate with adjunctive GnRHa during chemotherapy compared with chemotherapy alone, evidence of similar benefit in other cancer types is limited.^14^

For those undergoing radiotherapy, shielding of the pelvic organs is an easy and noninvasive way to minimize the gonadotoxic effect, although its effectiveness can be variable depending on the radiation field and scatter. An alternative, although still considered experimental, is uterus transposition. This surgical procedure involves temporarily moving the uterus outside the radiation field to preserve its function, and some live births have been reported following this technique.^45^, ^46^ Additionally, intensity‐modulated radiation therapy and proton radiotherapy can deliver energy more precisely to the tumor, reducing radiation exposure and potential damage to healthy tissue.^47^, ^48^

## 6 FERTILITY‐SPARING SURGERY TREATMENT

Fertility‐sparing surgery enables patients to retain their ability to conceive after a gynecological cancer diagnosis using their own uterus and gametes. Adequate staging is vital to determine whether it can be performed safely.

In patients presenting with an adnexal mass, thorough surgical staging includes peritoneal washings, unilateral salpingo‐oophorectomy (with cystectomy considered for borderline ovarian tumors), infracolic omentectomy, and peritoneal biopsies. When indicated, retroperitoneal lymphadenectomy is also performed. Surgery can be safely offered to patients with borderline ovarian tumors, malignant ovarian germ cell tumors (across all stages), granulosa cell tumors (FIGO Stage IA to IC), Sertoli–Leydig cell tumors (grade 1 and 2, FIGO Stage IA), and low‐grade (serous, endometrioid, and expansile mucinous) epithelial ovarian cancers (EOC) confined to FIGO Stages IA to IC. It is also considered safe for early‐stage high‐risk histologies, such as clear cell tumors (FIGO Stages IA to IC) and high‐grade (serous, infiltrative mucinous) EOC (FIGO Stage IA).^22^, ^49^, ^50^ The risk of relapse is determined more by tumor biology than by the decision to undertake fertility‐sparing surgery.

For early‐stage uterine cervical cancers, fertility‐sparing surgery is considered safe for early‐stage cancers of squamous cell carcinoma and adenocarcinoma measuring ≤2 cm in diameter with tumor depth invasion of ≤10 mm.^21^, ^22^ Aside from FIGO Stage IA1 disease without lymphovascular space invasion (LVSI), pelvic lymph node staging should confirm early‐stage, cervix‐confined disease with a low recurrence risk. While the majority of cervical cancers are HPV‐related, approximately 5%–11% are non‐HPV‐related adenocarcinomas, which can have a worse prognosis and may not be suitable for fertility‐sparing surgery.^51^

The international SENTIX trial recently indicated that sentinel lymph node (SLN) ultrastaging—a meticulous pathological examination involving serial sectioning and immunohistochemistry to detect micrometastases—had a high sensitivity exceeding 90% for identifying lymph node involvement in apparent early‐stage cervical cancer.^52^ Consequently, systematic lymphadenectomy is reserved for patients where SLNs cannot be detected.^49^

The ConCerv trial demonstrated that conization can be safely performed in patients with FIGO Stage IA2–IB1 LVSI‐negative tumors, yielding a low recurrence rate of 2.4%.^53^ A trial evaluating physical function and quality of life after nonradical surgery for early‐stage cervical cancer reported similar outcomes; among 68 patients with available survival data, 4.4% had recurrent disease after a median follow‐up of 37 months.^54^ This population overlaps with FIGO Stage IB1 LVSI‐positive tumors, which are traditionally managed with radical trachelectomy. Depending on surgical expertise, trachelectomy can be performed abdominally or vaginally. However, trachelectomy poses challenges for conception and increases the risk of preterm delivery; thus, cervical cerclage is usually recommended during the procedure or prior to conception.

For tumors measuring 2–4 cm, neoadjuvant chemotherapy (e.g. 3–4 cycles of carboplatin–paclitaxel) followed by conization has demonstrated favorable obstetric outcomes in retrospective studies. The ongoing international multicenter CoNteSSa‐NeoCon‐F trial (NCT04016389) aims to corroborate the feasibility of fertility preservation in this patient population.^55^

Considering endometrial cancer, fertility‐sparing treatment can be offered to patients diagnosed with endometrial atypical hyperplasia and endometrioid endometrial cancer grade 1 without myometrial invasion and may be considered in those with grade 2 disease.^56^ Transvaginal ultrasound and/or pelvic magnetic resonance imaging should be performed preoperatively to exclude myometrial invasion and adnexal involvement. Hysteroscopic endometrial resection followed by progestin‐based treatment is the most effective approach. Progestin‐based therapy consists of orally administered megestrol acetate 160–320 mg/day and/or a levonorgestrel intrauterine device at a dose of 52 mg for at least 6 months. Diagnostic hysteroscopy with endometrial biopsies is performed every 3 months to determine treatment response. After achieving two consecutively negative endometrial biopsies with an interval of at least 3 months, pregnancy might be encouraged. A large meta‐analysis reported a treatment response rate of 80%, with 35% of patients having disease recurrence during follow‐up.^57^ Patients with mismatch repair deficient (MMRd) tumors have a lower remission rate and are more likely to have disease recurrence.^58^ Live birth was reported for 69% of patients and two‐thirds were achieved through ART.^57^ A hysterectomy is recommended after completion of childbearing, if there is no response following 6–12 months of hormonal treatment, as well as in cases of disease recurrence or progression.^56^

## 7 FERTILITY TREATMENT OPTIONS AFTER CANCER

Fertility loss is nearly as significant a concern for reproductive‐aged women diagnosed with cancer as survival itself.^59^ Initial fertility assessments after cancer treatment typically include serum levels of FSH, estradiol, and AMH.^5^, ^35^ Ideally, transvaginal ultrasound to assess antral follicle count provides insight into ovarian reserve and reproductive potential. For male survivors, a semen analysis is the standard evaluation.

Clinicians should adopt a holistic approach to counseling, considering factors such as patient age, initial diagnosis, medical history, body mass index, life expectancy, treatment modalities, pregnancy‐associated risks, and reproductive history. All pregnancies achieved following cancer diagnosis and treatment require high‐risk obstetric surveillance.^29^

### 7.1 Spontaneous pregnancy

Pregnancy rates among cancer survivors are lower than those of age‐matched peers.^60^ When couples achieve spontaneous pregnancy, an individualized approach regarding the interval between treatment and conception is essential. Current evidence lacks consensus on the most effective interval; some guidelines recommend postponing pregnancy for 6–12 months, while others suggest a delay of up to 2 years in case there is early recurrence during pregnancy.^29^, ^61^ Pregnancy should not be attempted during active cancer therapy, as short intervals between treatment and conception are associated with increased risks of preterm birth and low birth weight.^62^, ^63^ One study indicated that survivors who delayed conception for 1 year after starting chemotherapy (without radiation), 2 years after starting chemotherapy (with radiation), and 1 year after cervical cancer diagnosis experienced the lowest risks of preterm birth.^64^ Survivors of childhood or adolescent cancer are less likely to achieve pregnancy and face higher risks of severe maternal morbidity and preterm birth.^64^ Delaying conception is also advised to allow time for detection of recurrences (as these patients may not be suitable for fertility‐sparing surgery or treatment), to minimize potential toxic effects of treatment on gametes, and to mitigate complications from immunosuppression, anemia, cardiovascular effects, physical stress, or insufficient weight gain during pregnancy.^61^ A multidisciplinary approach and consultation with the treating team are crucial for optimizing survival likelihood while maintaining the chance of pregnancy.^63^

### 7.2 Assisted reproductive technologies

For patients who have undergone gamete or embryo cryopreservation, pregnancy may be achieved through ART, typically IVF, or intracytoplasmic sperm injection (ICSI). For those with ovarian insufficiency or azoospermia after cancer treatment, donor gametes or embryos are viable options. Conversely, patients undergoing ovarian tissue autotransplantation have reported live birth rates of 19%–32%.^5^

Regarding success rates, chemotherapy diminishes ovarian reserve and response to ovulation induction in ART.^65^ Studies show that conception rates are higher when IVF is initiated before cancer treatment rather than after and when freeze durations are shorter.^66^ Success rates for frozen sperm and embryos are comparable to those of age‐matched noncancer patients. The outcomes of thawed and injected oocytes depend on female age, with younger females achieving the highest success rates.

It is critical for fertility specialists to adopt a tailored treatment approach, weighing the benefits and risks of ART. Many countries provide at least one cycle of state‐funded ART, contingent on couples meeting eligibility criteria.

### 7.3 Surrogacy

For women with adequate ovarian reserve who are unable to carry a pregnancy due to surgical or radiation‐induced uterine damage, traditional or gestational surrogacy may offer alternative pathways. In such cases, oocytes can be retrieved and subsequently undergo IVF, with the resulting embryos transferred to a gestational surrogate. Laws governing surrogacy vary significantly between countries; thus, clinicians must consider the legal framework within their own jurisdictions. Some countries permit only altruistic surrogacy. Couples exploring international surrogacy should also be aware of the significant financial implications.

### 7.4 Adoption

Adoption is another option for cancer survivors. The adoption process can take from 1 to 5 years, with varying regulations on foreign adoption depending on the country. Couples may also explore foster care adoption. Adoption agency policies may deem families ineligible based on factors such as medical history, life expectancy, age, sexual orientation, and marital status.

## 8 OPTIMIZING GENERAL HEALTH AND PREPREGNANCY COUNSELING

Beyond fertility preservation, comprehensive care for cancer survivors planning a family requires optimizing general health at least within the year prior to conception, which can be optimized by thorough prepregnancy counseling. This holistic approach aims for the best possible outcomes for both patient and offspring.^29^ Key considerations include:

- *Organ function assessment:* Cardiac (echocardiography for cardiotoxic exposures), endocrine (thyroid function tests for neck/brain radiation or alkylating agents), respiratory (pulmonary function tests for bleomycin/chest/total body irradiation), renal, and hepatic function tests.
- *Thrombotic risk:* Assessment of thrombotic event history and individualized thromboprophylaxis planning.
- *Nutritional optimization:* Nutritional counseling is paramount, given the impact of cancer and its treatment on absorption and metabolic health. The FIGO Nutrition Checklist can guide discussions for optimal maternal and fetal well‐being.
- *Psychological well‐being*: Address emotional well‐being and offer access to mental health services.
- *Cancer‐specific considerations:* Tailored counseling, such as tamoxifen or trastuzumab treatment duration for breast cancer survivors, or cervical length monitoring after cervical cancer treatment.
- *Genetic counseling:* Offer genetic counseling for patients with hereditary cancer syndromes or those with treatment‐induced genetic changes to assess risk to offspring.
- *Vaccination and conception timing:* Ensure updated vaccine prophylaxis.

## 9 CLINICAL CASE EXAMPLES: FERTILITY PRESERVATION IN GYNECOLOGICAL MALIGNANCIES

Table 3 summarizes two distinct clinical case studies, illustrating important considerations for fertility preservation in patients with gynecological malignancies. These cases highlight the complex decision‐making process and the role of multidisciplinary boards in guiding treatment and fertility preservation strategies.

**TABLE 3:** Clinical case studies of fertility preservation in gynecological malignancies.

Feature	Case 1: Ovarian clear cell carcinoma	Case 2: Cervical cancer
Patient	37‐year‐old, nulliparous woman	29‐year‐old, nulliparous woman
Presentation/diagnosis	Two ovarian cysts suspicious for endometriosis found during IVF Following bilateral ovarian cystectomy: clear cell carcinoma in the right ovary; left cyst contained endometrial stromal fragments and hemorrhagic ovarian tissue. Cytology of free abdominal fluid: proteinaceous material and inflammatory cells	Squamous cell carcinoma of the cervix Transvaginal ultrasound and MRI revealed a 20‐mm tumor, corresponding to FIGO Stage IB1
Treatments proposed by requesting physician	Fertility preservation options after treatment were the primary subject of the consultation	Three options were proposed: Radical trachelectomy with pelvic lymphadenectomy (noting a 6–8‐mm distance from the internal os poses a recurrence risk) Sentinel lymph node procedure and pelvic lymphadenectomy, followed by neoadjuvant chemotherapy and trachelectomy if lymph nodes are negative Cryopreservation of oocytes, followed by radical hysterectomy with ovarian preservation and transposition to the upper lateral abdomen
Advisory Board on Cancer, Infertility, and Pregnancy (ABCIP) recommendation/consensus	Consensus was to proceed with comprehensive surgical staging including: ⚬ Removal of the right ovary ⚬ Omentectomy ⚬ Pelvic and para‐aortic lymphadenectomy ⚬ Curettage ⚬ Biopsy of the contralateral ovary if abnormal morphology is present	Recommended the sentinel lymph node procedure and pelvic lymphadenectomy If lymph nodes were negative, follow with neoadjuvant chemotherapy Advised a large cone biopsy instead of trachelectomy (due to low risk of parametrial involvement)
Fertility preservation	Considered for early‐stage disease (FIGO Stage I); not recommended for advanced stage (FIGO Stage II+)	Cryopreservation of oocytes was deemed unnecessary unless (chemo)radiation therapy anticipated Suggested an abdominal cerclage for future pregnancy
Key reflection	Surgical staging is crucial before finalizing a fertility preservation plan The risk of recurrence is primarily influenced by the tumor's biological behavior Preservation of the uterus and contralateral ovary is a lesser factor in recurrence risk compared with tumor biology	Patients with smaller cervical tumors may benefit from less radical surgery Less radical surgeries (e.g. large cone conization or simple trachelectomy) are suitable, particularly for tumors without deep stromal invasion, lymphovascular space invasion, or lymph node metastasis Future pregnancy planning should be an integral part of treatment decisions

Abbreviations: IVF, in vitro fertilization; MRI, magnetic resonance imaging.

The Advisory Board on Cancer, Infertility, and Pregnancy (ABCIP) is a specialized multidisciplinary board that offers guidance on fertility preservation strategies for cancer patients. Both cases presented here were referred to the ABCIP for expert consultation.

## 10 FUTURE RESEARCH DIRECTIONS

The intersection of cancer and fertility management is dynamic and requires ongoing research to address gaps and challenges. Priorities include:

- **Early diagnosis and risk prediction:** Develop better tools to assess individual infertility risk based on cancer type, treatment regimen, and reproductive biomarkers (e.g. AMH, FSH).
- **Fertility‐sparing interventions:** Refine medical and surgical strategies to preserve fertility without compromising oncologic outcome, particularly in early‐stage or rare cancers.
- **Gonadal protection during treatment:** Explore new pharmacologic and technical strategies to reduce the risk of treatment‐induced sterility.
- **Innovations in cryopreservation:** Advance techniques such as in vitro maturation and tissue cryopreservation, and evaluate the long‐term viability and use of stored reproductive material.
- **Post‐treatment fertility outcomes:** Conduct longitudinal studies on pregnancy rates, reproductive health, and offspring outcomes following cancer therapy and fertility preservation.

## 11 LEGAL AND POLICY CONSIDERATIONS

The implementation of effective fertility preservation in cancer care requires supportive legal and political infrastructure. Key recommendations include:

- **National frameworks**: Establish national policies that define infertility prevention as a core component of oncology care, including timely access to fertility preservation.^5^, ^16^, ^17^
- **Equity of access**: Enact legislation to ensure publicly funded or insurance‐covered access to fertility preservation for medically indicated cases, including for adolescents and individuals with rare cancers.
- **Regulatory oversight**: Develop clear legal guidance on the use, ownership, and disposition of cryopreserved gametes and embryos, especially in cases of death, separation, or loss of capacity.^16^
- **Ethical governance**: Align national laws with international ethical standards regarding surrogacy, use of donor gametes, and access for individuals with hereditary cancer syndromes.^19^
- **National registries and data collection**: Support centralized tracking systems to monitor utilization, outcomes, and safety of fertility preservation and subsequent pregnancies in cancer survivors, guiding future policy and funding decisions.^67^

Table 4 summarizes key recommendations for both clinicians and health policy makers to further advance oncofertility care.

**TABLE 4:** Summary of key recommendations for clinicians and health policy makers.

Area	Clinicians	Policy makers
1. Early and comprehensive counseling	Initiate timely, individualized discussions at diagnosis, prior to cytotoxic therapy Emphasize the time‐sensitive nature of fertility preservation Educate on gonadotoxicity risks from conventional (e.g. alkylating agents, pelvic radiation) and emerging therapies (e.g. PARP inhibitors, TKIs) Provide detailed information on standard (oocyte/embryo cryopreservation, sperm banking, ovarian tissue cryopreservation GnRHa) and experimental options (IVM, TESE, donor gametes, PGT‐M) Refer to psychosocial and financial support services Counsel on relevant legal/ethical considerations (e.g. material ownership)	Mandate oncofertility counseling as a standard component of cancer care Develop national protocols ensuring early referral to fertility specialists
2. Integration of fertility preservation	Integrate fertility preservation into standard oncologic pathways Coordinate multidisciplinary teams (oncology, reproductive medicine, genetics, mental health)	Recognize fertility preservation as a national healthcare priority Implement policies for equitable access, addressing financial and geographic barriers Standardize and require insurance coverage for medically indicated fertility preservation when relevant
3. Research and knowledge gaps	Support research on gonadotoxicity of novel therapies (e.g. ICIs, ADCs) Study long‐term safety, washout periods, and pregnancy outcomes Stay updated on emerging fertility preservation techniques (e.g. IVM)	Allocate dedicated funding for oncofertility research and long‐term outcome studies Support prospective studies to define safe conception windows after treatment Promote international collaboration for data and guideline development
4. Clinical practice and management	Apply fertility‐sparing surgery where indicated, ensuring appropriate staging Advise on optimal conception timing after treatment Provide high‐risk obstetric care during pregnancies following cancer Tailor ART based on patient needs and legal context	Publish and disseminate evidence‐based clinical guidelines Support oncofertility training for healthcare providers
5. Patient support and accessibility	Offer transparent cost information and assist with financial navigation Provide ongoing psychosocial support Support exploration of alternative family‐building options (adoption, surrogacy)	Create funding mechanisms or subsidies to reduce financial barriers Develop national registries to monitor survivor fertility outcomes Lead public awareness efforts on fertility preservation in oncology

Abbreviations: ADC, antibody–drug conjugate; AMH, anti‐Müllerian hormone; ART, assisted reproductive technologies; GnRHa, gonadotropin‐releasing hormone agonist; ICI, immune checkpoint inhibitor; IVF, in vitro fertilization; IVM, in vitro maturation; PARP, poly (ADP‐ribose) polymerase; PGT‐M, preimplantation genetic testing for monogenic disorders; TESE, testicular sperm extraction; TKI, tyrosine kinase inhibitor.

## 12 CONSIDERATIONS FOR LOW‐ AND MIDDLE‐INCOME COUNTRIES

Fertility preservation in low‐ and middle‐income countries faces distinct challenges tied to resource limitations and sociocultural factors. Affordable options, including low‐cost cryopreservation and simplified ovarian tissue banking, should be supported by regional fertility centers. Increasing awareness among providers and patients, especially in rural areas, is crucial for improving access.^67^, ^68^

Advocacy for national policies that prioritize fertility care, along with training programs for providers through academic partnerships, can drive meaningful progress. Any newly established national oncology program should allocate dedicated funding for the development of expertise in fertility preservation, ensuring it becomes an integrated part of comprehensive cancer care.

Culturally sensitive programs and telemedicine can extend care to underserved populations. Global collaboration can further enhance knowledge‐sharing, resource distribution, and research efforts to ensure fertility preservation practices for cancer patients worldwide.^68^

## AUTHOR CONTRIBUTIONS

All authors contributed to the design, planning, conduct, analysis, and manuscript writing.

## FUNDING INFORMATION

None.

## CONFLICT OF INTEREST STATEMENT

The authors have no conflicts of interest.

## Data Availability

Data sharing is not applicable to this article as no new data were created or analyzed in this study.
